# Hydrodynamic and hydro acoustic analysis of marine propeller in off design flow conditions

**DOI:** 10.1371/journal.pone.0320435

**Published:** 2025-03-24

**Authors:** Ammar Nazeer, Niaz Bahadur Khan, Emad Uddin, Mohammed Jameel, Omar Al-Abbasi, Qian Wu, Adnan Munir, Hanzla Shahid

**Affiliations:** 1 Department of Mechanical Engineering, School of Mechanical & Manufacturing Engineering (SMME), National University of Sciences and Technology (NUST), Islamabad, Pakistan; 2 Mechanical Engineering Department, College of Engineering, University of Bahrain, Isa Town, Bahrain; 3 Department of Civil Engineering, College of Engineering, King Khalid University, Abha, Saudi Arabia; 4 School of Aeronautics and Astronautics, University of Electronic Science and Technology of China, Chengdu, China; 5 School of Engineering, Design and Built Enviroment, Western Sydney University, Penrith, New South Wales, Australia; 6 Department of Mechanical Engineering, Khalifa University of Science and Technology, Abu Dhabi, United Arab Emirates; NED University of Engineering and Technology, PAKISTAN

## Abstract

This study provides a comprehensive analysis of the INSEAN E-779A four-bladed marine propeller, addressing both hydrodynamic and hydroacoustic aspects. Employing unsteady Reynolds-Averaged Navier-Stokes (RANS) simulations with the k-ω SST turbulence model for hydrodynamics and the Ffowcs Williams-Hawking acoustic analogy for acoustics. A wide range of operational conditions are examined by varying the advance ratio from 0.6 to 0.9 and flow incidence angle from 0 to 40 degrees. Computation of hydrodynamic coefficients across spatial directions provides insight into loading impacts on performance. Significant efficiency reductions are observed, such as a decrease from 64% to 28% at 40 degrees for an advance ratio of 0.88. Directional acoustic pressure distributions reveal notable variance, including a 19–31% change when reducing the advance ratio to 0.6 at 40 degrees incidence. Fast Fourier Transform (FFT) analysis of acoustic signals highlights dominant frequencies and acoustic signature changes downstream of the propeller. Notably, peak Sound Pressure Level (SPL) values at specific locations in the propeller’s wake demonstrating sensitivity to flow conditions. The investigation extends to near-field and far-field acoustic signatures, contributing to a comprehensive understanding of how acoustic behavior evolves with distance.

## Introduction

Marine propellers are important components of vessel propulsion systems. They typically consist of multiple blades attached to a central hub, connected to diesel engines via a drive shaft to provide thrust. Even small improvements to propeller hydrodynamic efficiency can significantly reduce operating costs. The acoustic characteristics of marine propellers hold substantial importance. Unwanted propeller noise can significantly affect passenger comfort, particularly on commercial vessels. Additionally, in naval and submarine operations, propeller acoustic signatures are vital indicators. To minimize detection by other vessels, it is crucial to operate in flow conditions that reduce noise. Furthermore, propeller acoustic noise is a major contributor to underwater noise pollution, adversely affecting marine ecosystems. Extensive research has been conducted to study marine propeller behavior, covering various aspects of hydrodynamics, acoustics, and optimization techniques.

Bagheri et al. [1] conducted a comprehensive analysis of a five-bladed marine propeller. The experimental and numerical study revealed the importance of operating pressure and rotational speed in inducing cavitation, with a notable increase in Sound Pressure Level (SPL) observed when reducing pressure from 90 Kpa to 70 Kpa. Dubbioso et al. [2] investigated the complex hydrodynamics of marine propellers operating under extreme off-design conditions, employing unsteady Reynolds-averaged Navier–Stokes (URANS) equations coupled with a dynamically overlapping grid approach. Their study on the CNR-INSEAN E779A propeller model highlighted the influence of oblique flow conditions on hydrodynamic loads and wake dynamics. This work provided significant insights into the pressure distribution on propeller blades and the hub, especially during severe maneuvers, and emphasized the challenges in accurately predicting performance at high incidence angles. Karsilnikov et al. [3] analyzed unsteady forces on propeller blades using RANS simulations. Their study aimed to assess the capabilities of unsteady RANS models in predicting forces and pressure distribution, particularly for open and podded propellers. Shamsi and Abbasi et al [4, 5] investigated propeller performance in oblique flow conditions using Multiple Reference Frame (MRF) and sliding mesh techniques, focusing on open-water propellers. Their study highlighted the advantages of the sliding mesh technique for analyzing the dynamic nature of propellers. In the domain of hydrodynamics and hydroacoustics, Bagheri et al. [6] utilized the Finite Volume Method (FVM) to examine the hydrodynamic behavior of marine propellers. They explored performance curves and acoustic predictions under both cavitating and non-cavitating conditions, emphasizing the influence of cavitation on sound levels. Kim et al. [7] examined the acoustic behavior of non-cavitating propellers in nominal and effective wake scenarios. Their observations revealed elevated Sound Pressure Levels (SPL) in nominal wake conditions compared to effective wake conditions. Additionally, they found minimal influence of propeller rake on both SPL and acoustic energy. Felli et al. [8] demonstrated the innovative use of tomographic PIV for analyzing marine propeller hydrodynamics and hydroacoustics. The hydrodynamic analysis revealed detailed vortex structures in the propeller wake, with a focus on vorticity transport mechanisms. Additionally, tomographic PIV was combined with Powell’s acoustic analogy for the hydroacoustic analysis, accurately capturing the source terms. The results showed that the tip vortex perturbation is the dominant nonlinear contributor to far-field noise in non-cavitating flow conditions.

Yao et al. [9] contributed to the field by investigating hydrodynamic performance across various combinations of oblique angles and advance ratios. Using OpenFOAM and RANS techniques with a sliding grid approach, Yao’s work provided valuable insights into the nuances of propeller behavior under different condition.

Tani et al. [10] investigated pressure pulses and radiated noise from different propeller designs for fast crafts. They considered forward and backward rake propellers and found cases where hydrodynamic loading dominated pressure pulses over cavitation effects. Ebrahimi et al. [11] examined strategies to reduce propeller noise, including blade coating, modern designs, hub modifications, and ducted propellers. Their study assessed how geometric parameters influenced noise levels and operational ranges, culminating in an algorithm for optimal propeller design. Felli et al [12] conducted groundbreaking research on the mechanisms of noise generation and emission from naval propellers, particularly focusing on the integration of direct pressure fluctuation measurements and advanced flow diagnostics. Utilizing techniques such as Tomographic Particle Image Velocimetry (PIV) combined with Powell’s acoustic analogy, they identified the dominant contributions of tip vortices and hydrodynamic interactions in propeller noise generation under non-cavitating conditions. Ianniello and Testa [13] explored differences between aeronautical and marine device acoustic fields. He found that marine propeller acoustics are inherently nonlinear due to rotating sources and diverse rotational speeds, challenging assumptions about negligible nonlinear contributions at low subsonic speeds. Pennings et al. [14] analyzed the acoustic signature of a cavitating vortex by synchronized simultaneous visualization of cavitation pattern and noise measurements in order to gain a deeper insight into propeller tip vortex cavitation. Ianniello [15] studied propeller acoustic behavior using scaled ship models and unsteady RANS with FW-H acoustic analogy. They analyzed twin propellers in non-cavitating, open water conditions, considering thickness, loading components, quadrupole terms, and sound scattering due to ship hull interactions, highlighting the effectiveness of acoustic analogy.

Cianferra et al. [16] investigated hydrodynamic noise from marine propellers using LES and FW-H acoustic analogy. Their analysis of non-cavitating, open water conditions via Detached Eddy Simulations (DES) revealed aspects of noise generation, emphasizing the importance of the quadrupole term in this context. Mousavi et al. [17] studied the acoustic behavior of the propeller using LES and FW-H models. They conducted directivity analysis and compared them with the flow field generated by the propeller. Li et al. [18] characterized Underwater Radiated Noise (URN) of a ship using full-scale measurements, model testing, and CFD, demonstrating good agreement for sheet cavitation and tip vortex cavitation (TVC). The CFD accurately predicted tonal noise and pressure pulses but under-predicted broadband noise, particularly in the 50–112 Hz range, with a maximum deviation of 28 dB. The study highlights the method’s potential despite challenges in capturing high-frequency broadband noise. Keller et al. [19] studied the flow field of a five-bladed marine propeller operating at design conditions using large eddy simulations to calculate the resulting far-field sound. The results of three acoustic formulations are compared Ffowcs-Williams and Hawkings (FW-H), Curle acoustic analogy and a point-force dipole model. Lidtke et al. [20] investigated the acoustics of propellers in both cavitating and non-cavitating conditions using the porous FW-H method coupled with a viscous RANS solver. Their goal was to understand the sensitivity of the analogy to the definition of porous data surfaces and key simulation parameters, such as time step and grid resolution. Wang et al. [21] explored the sound field properties of non-cavitating marine propellers. They employed a hybrid method combining the FW-H analogy with the Boundary Element Method (BEM) to study the decay rate of sound with respect to distance from the propeller center. This study also examined two different formulations of the FW-H analogy: permeable and direct. Dubbioso et al [22] conducted a study on the hydro-acoustic performance of a marine propeller behind a ship. They focused on a five-bladed propeller and analyzed its behavior in the ship’s wake. The study highlighted the significant influence of ship motion-induced velocities on the propeller’s inflow and its subsequent impact on noise emissions. To gain a better understanding of this complex interplay between hydrodynamics and acoustics in practical marine settings, they used a hybrid approach combining Computational Fluid Dynamics (CFD) simulations for inflow with a blade element momentum theory solver (BEMT) for noise source calculations.

The objective of this study is to comprehensively analyze the impact of advance ratio (J) and angle of attack on hydrodynamic and hydroacoustic performance of marine propellers using an unsteady RANS model with the k-ω SST turbulence model and the Ffowcs-Williams Hawkings (FW-H) acoustic analogy.

The choice to utilize the SST k-omega turbulence model over more advanced schemes like Detached Eddy Simulation (DES) or Large Eddy Simulation (LES) was primarily driven by computational efficiency and practicality. DES and LES approaches typically require substantially larger computational resources and longer simulation times due to their higher fidelity in resolving turbulent flow structures. However, for propeller design purposes where multiple simulations are often required to explore various operating conditions, the computational cost associated with DES or LES becomes prohibitively expensive. The SST k-omega model strikes a balance by offering reasonable accuracy in capturing turbulence effects while remaining computationally feasible for conducting parametric studies and optimization tasks within a reasonable timeframe. Therefore, the SST k-omega model is opted to achieve a practical compromise between computational cost and simulation accuracy in their propeller hydrodynamic analysis.

## Numerical models and methodology

### Hydrodynamic and acoustic modelling

Pressure-based transient analysis is performed using *k-ω SST* turbulence model, coupled with Ffowcs Williams and Hawking (FW-H) acoustic model to predict the propeller’s hydro-acoustic performance. The solution method includes the SIMPLE scheme for pressure-velocity coupling, a second-order upwind scheme for diffusive and convective terms, and a second-order implicit method for the transient formulation. Acoustic analysis was executed using Ffowcs William and Hawking method.

### Geometry and mesh detail

**Fig 1:**
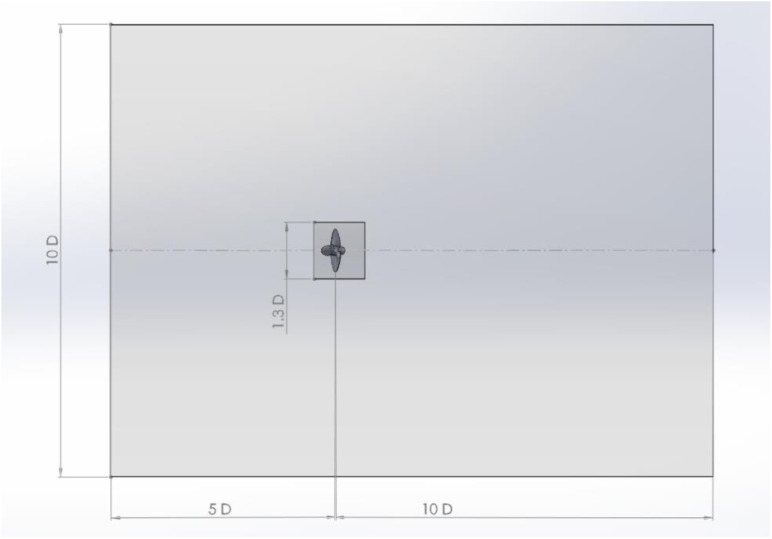
Details of the computational domain.

**Fig 2:**
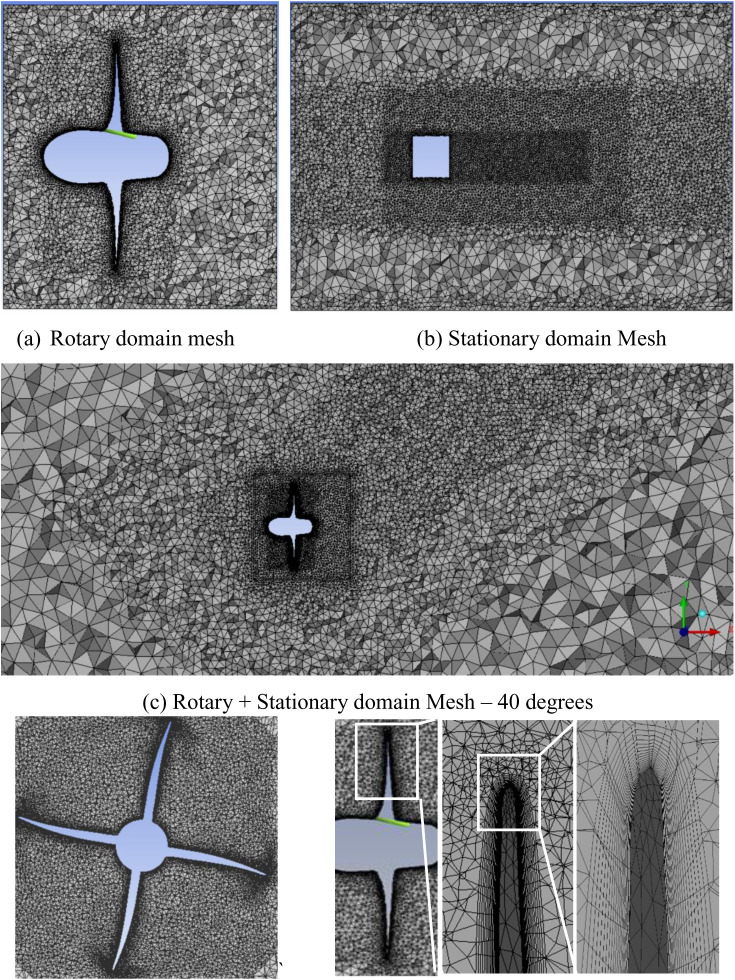
Meshing of rotary and stationary domain with X-sectional and boundary layer details.

3-D modeling of the INSEAN E-779A propeller was carried out in SolidWork. The computational domain was divided into two parts: a rotating domain housing the propeller (as shown in Figs 1 and 2), and a stationary circular domain encompassing the inlet and outlet boundaries. The stationary domain was tilted at different angles to control the propeller’s angle of attack. Both the rotating and stationary domains were meshed separately using ICEM meshing software and then combined within Fluent.

An unstructured hybrid mesh with a wall Y + factor ranging from 30 to 150 was generated. The first wall layer had a thickness of 0.001 mm and was stretched to include 10–15 layers with a 1.05 ratio. Fig 2 shows a closer view of the mesh, focusing on the inflation layers adjacent to the blade wall. The rotating domain consists of approximately 1.7 million cells, where similar mesh configuration settings were used for all other cases. However, the stationary domain was meshed independently for each case, resulting in cell counts ranging from 2.1 million to 2.6 million cells. This led to an overall cell count in the range of 4.8 million to 5.4 million cells. Table 1 presents the grid generation details for the mesh independence study. The meshes were evaluated against experimental data for thrust and torque coefficients. Mesh 3 demonstrated superior suitability for the case studies.

**Table 1:** Details of Grid Generation for Mesh independence.

Mesh Resolutions				
	Mesh 1	Mesh 2	Mesh 3	Mesh 4
No. of Cells for Rotational Domain	1.2 millions	1.4 millions	1.7 millions	2.0 millions
% Error K_T_	7.2	2.3	0.6	0.3
% Error 10 K_Q_	8.3	3.1	1.9	1.4

Cases were run by rotating the propeller at a fixed speed of 25 rps, with variations in the advance ratio achieved by adjusting the inflow free stream velocity to the propeller. The overall summary of the numerical setup is shown in Table 2.

**Table 2:** Numerical simulation parameters summary.

Analysis type	Pressure based Transient
**Turbulence model**	k-ω SST
**Acoustic model**	FW-H
**Flow material**	Water
**Pressure – Velocity coupling**	SIMPLE
**Convective and Diffusive Discretization Terms**	Second order upwind
**Transient formulation**	Second order implicit
**Boundary conditions**	Blades and Hub = Wall with ω = 0 rpms and no slip Rotating domain = Sliding mesh motion (ω = 1500 rpms) Inlet velocity $u_{\infty }$ = 5 m/s Outlet = Pressure Outlet

## Results and discussion

### Hydrodynamic performance.

The hydrodynamic performance of the marine propeller was evaluated in terms of the thrust coefficient (K_T_), torque coefficient (K_Q_), advance ratio (J), and propulsive efficiency (η). These parameters are defined by the following set of equations:


$$K_{T}=\frac{T}{\left(\rho N^{2}D^{4}\right)}$$

$$K_{TX}=\frac{T_{X}}{\rho N^{2}D^{4}},K_{TY}=\frac{T_{Y}}{\rho N^{2}D^{4}},K_{TZ}=\frac{T_{Z}}{\rho N^{2}D^{4}}$$ (1)

$$K_{Q}=\frac{Q}{\rho N^{2}D^{5}}$$

$$K_{QX}=\frac{Q_{X}}{\rho N^{2}D^{5}}K_{QY}=\frac{Q_{Y}}{\rho N^{2}D^{5}}K_{QZ}=\frac{Q_{Z}}{\rho N^{2}D^{5}}$$ (2)

$$\eta =\frac{JK_{T}}{2\pi K_{Q}}$$

$$J=\frac{U_{\infty }}{ND}$$

where,

T =  Thrust of propeller, Q =  Torque of propeller, N =  Revolutions per second, D =  Diameter of Propeller, U_ ∞_ =  Free stream velocity, ρ =  Density of fluid

**Fig 3:**
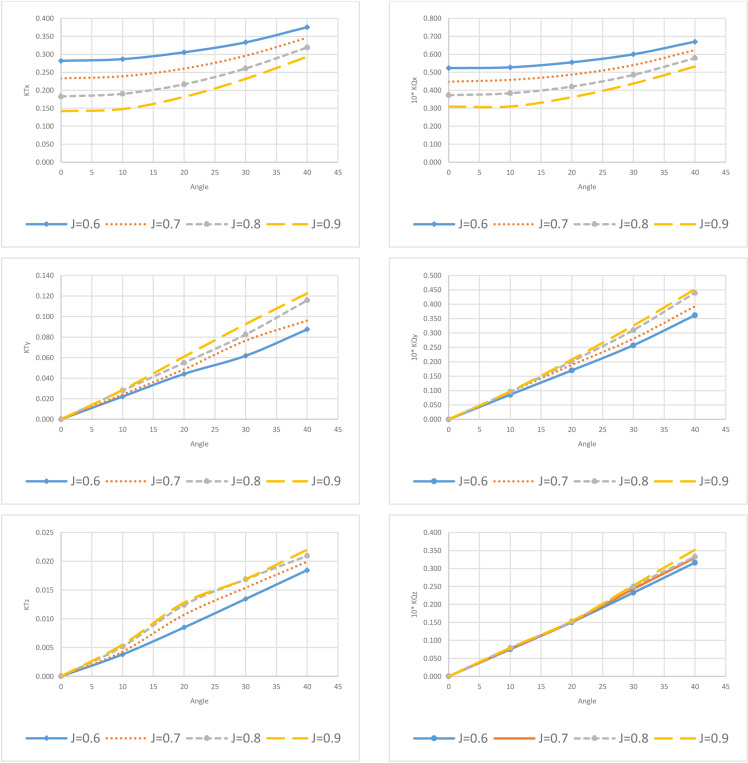
Thrust and torque coefficients graphs for J = 0.6–0.9 and θ =  0–40 degrees.

Being a highly symmetrical body, the propeller produces forces in the lateral direction, i.e., K_TY_, K_TZ,_ due to its asymmetric interactions with the flow field or incoming free stream. Since the propeller works as a screw by cutting the flow, these asymmetrical interactions result in lower Y and Z directional component values when operating at lower advance ratios resulting in higher loads on the propeller. It is noteworthy that hydrodynamic parameters exhibit an increasing trend with an increase in the angle of incidence to the propeller, which implies that there is more loading on the propeller to maintain the same advance speed. Fig 3 shows the thrust and torque coefficients for different values of J and angles.

**Fig 4:**
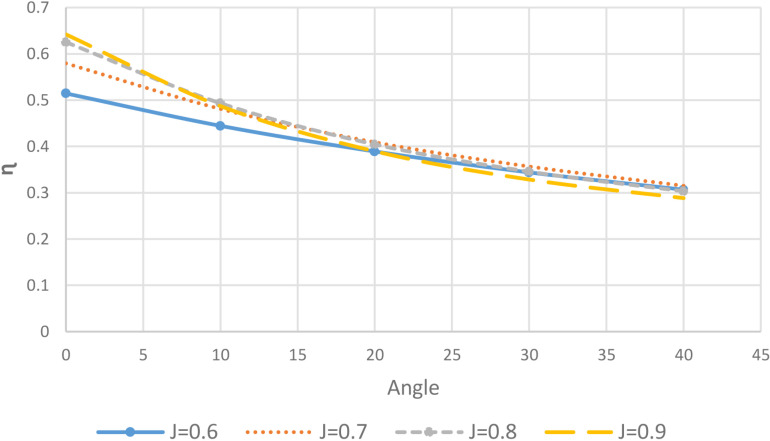
Efficiency of the Propeller for J = 0.6–0.9 and θ =  0 – 40 degrees.

As shown in Fig 4, the maximum decrease in efficiency occurred for the advance ratio of J = 0.88, dropping from 64% at 0 degrees to 28% at 40 degrees incidence angle. In comparison, for J = 0.6 the efficiency reduced less severely from 51% at 0 degrees to 40% at 40 degrees. Efficiency is defined as the ratio of thrust to torque coefficients, indicating the portion of input torque converted to useful thrust. The result indicates that reduced efficiency is primarily attributed to phenomena like flow separation and cavitation on the propeller blades, which increase blade loading. At the lower advance ratio of J = 0.6, the propeller is already operating in a more loaded state at 0 degrees incidence, as evident by the lower initial efficiency. Hence, increasing the angle of attack to higher values did not substantially impact efficiency due to the propeller’s existing high-loading conditions. However, at J = 0.88 the propeller is closer to its design point of optimal operation. As such, changing the incidence angle from 0 to 40 degrees had a much greater deteriorating effect on efficiency, causing it to decrease to half of the original value. This highlights the propeller’s sensitivity to flow deviations when operating near its peak performance condition.

**Fig 5:**
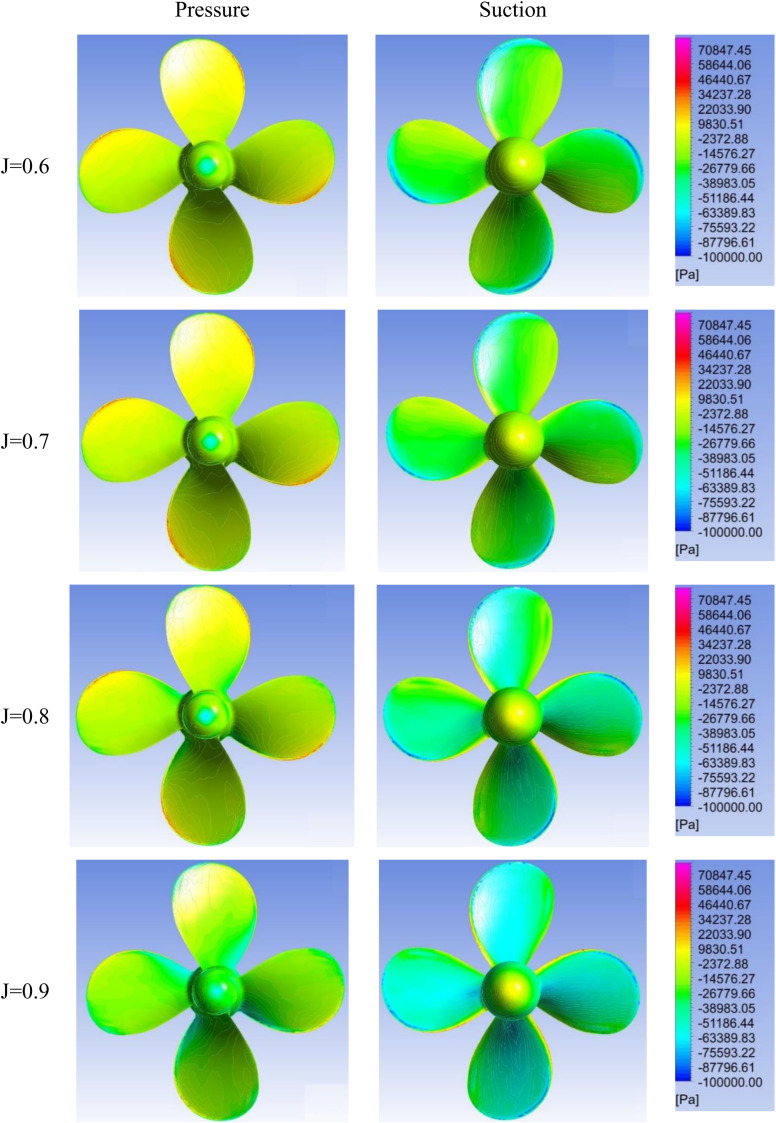
Pressure contours on suction & pressure side of propeller for J = 0.6–0.9 and θ =  0.

**Fig 6:**
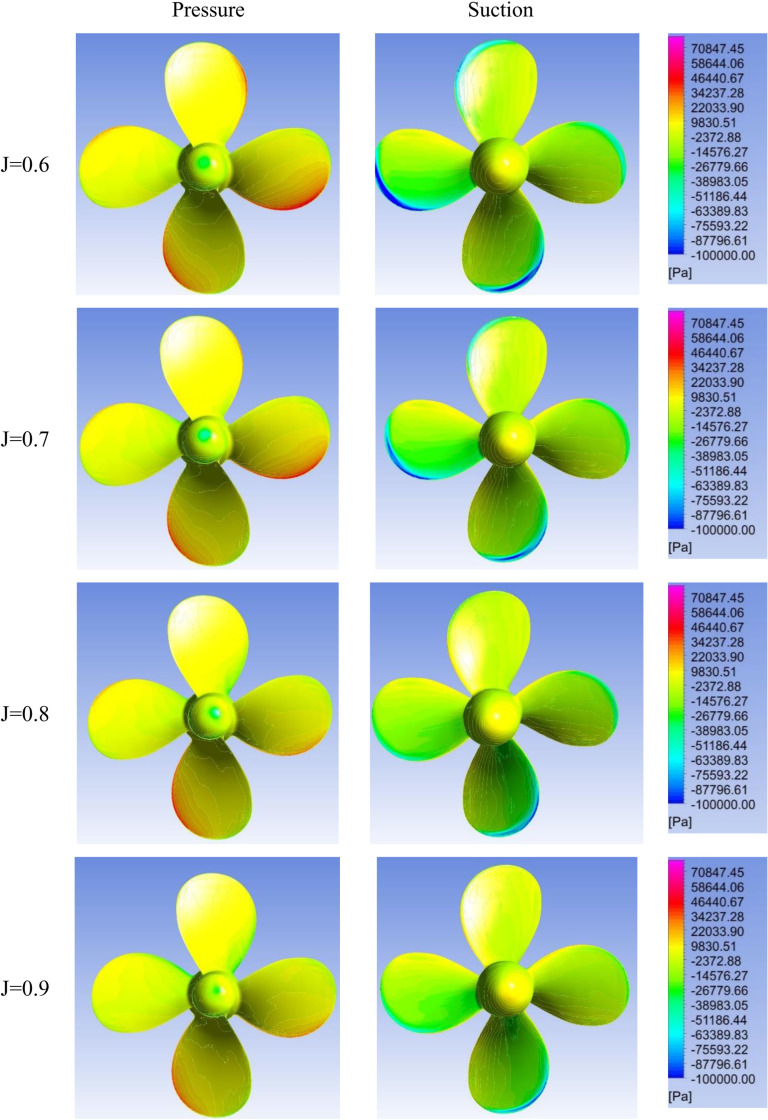
Pressure contours on suction & pressure side of propeller for J = 0.6–0.9 and θ =  20.

**Fig 7:**
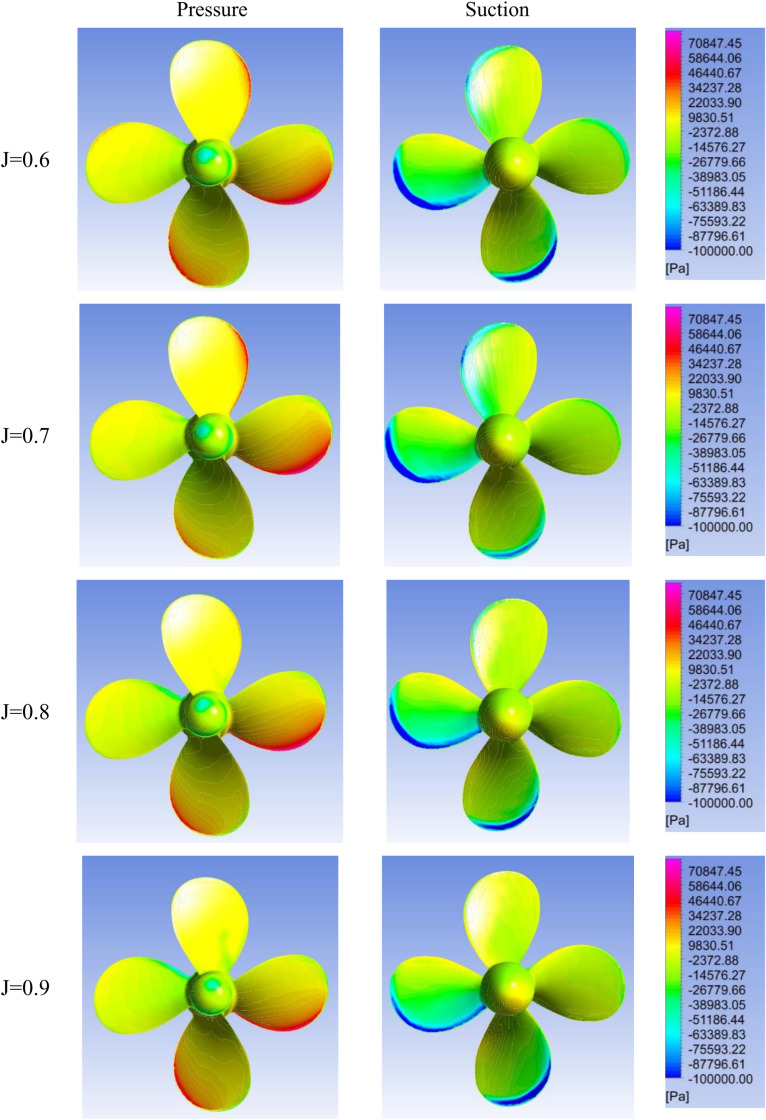
Pressure contours on suction & pressure side of propeller for J = 0.6–0.9 and θ =  40.

Fig 5–7 show contours on the suction and pressure sides of the propeller blades for varying advance ratios (J =  0.6–0.9) and incidence angles (θ =  0°, 20°, 40°). At 0° incidence, a uniform, symmetric loading is visible across all J indicative of design-like flow conditions. Comparatively, the propeller experiences noticeably higher loading at lower advance ratios of J = 0.6–0.7 for all incidence angles, attributable to the higher propeller RPM required to maintain ship speed against the oblique flow. The propeller loading also increases significantly with rising incidence angle for a given advance ratio. Moreover, highly asymmetric pressure distributions become visible on the propeller blades at 20° and 40° incidence.

**Fig 8:**
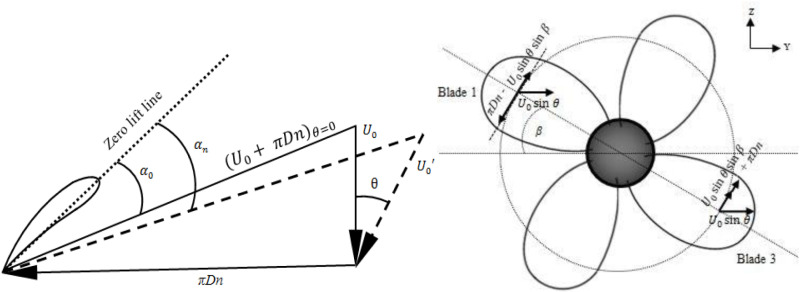
Vector representation of the difference in velocity across propeller blades.

The velocity vector representation in Fig 8 can explain the uneven pressure distribution across the propeller blades under oblique inflow conditions. As the flow enters the propeller at a skewed angle of attack, the velocity experienced by different blades is unequal, as depicted in Fig 8. This effect is clearly visible in the 40° angle of attack case (Fig 7), where two blades undergo much higher suction pressures and the other two undergo elevated pressure pressures due to the asymmetric velocities with propeller diameter 0.227 m in dimension. The resulting uneven velocity distribution leads to the skewed pressure profiles across the blades.

**Fig 9:**
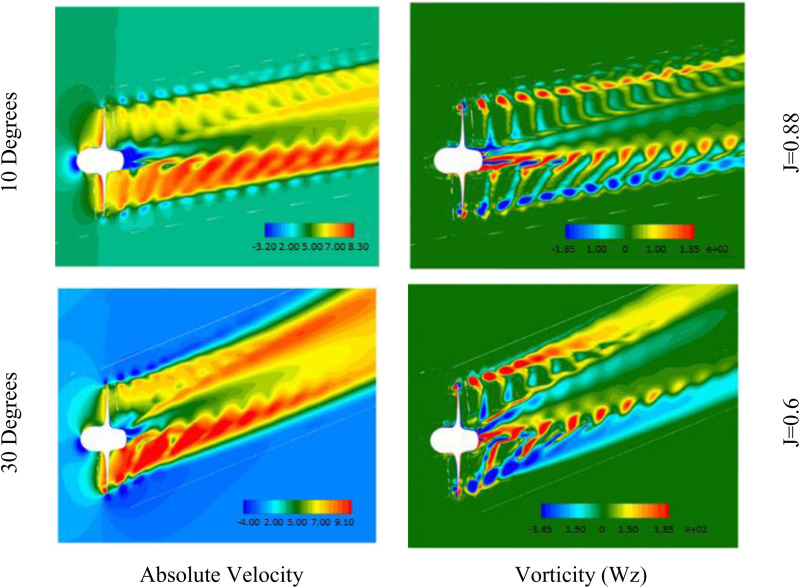
Absolute velocity and Vorticity contours in XY-plane.

Contours of absolute velocity and vorticity (Wz) in the XY-plane are presented in Fig 9. These contours provide further evidence of the increased loading experienced by the propeller at lower advance ratios and higher incidence angles. The increase load on the propeller is due to asymmetric interaction with the incoming flow field. At 30 degrees incidence, higher vorticity values are observed compared to 0 degrees, corresponding to greater blade loading. The J = 0.6 case also exhibits stronger vorticity in the propeller wake region compared to J = 0.88, indicating higher hydrodynamic forces.

The elevated vorticity at reduced advance ratio and increased incidence angle agrees with trends seen in the hydrodynamic coefficients and pressure contours. Fig 9 complements these analyses by spatially resolving details of the flow field around the propeller. Collectively, Figs 5–7 and 9 provide a visual demonstration of how propeller loading amplifies during off-design operating conditions through changes in local velocities and vortex structures.

### Acoustic analysis

Acoustic analysis of propeller was carried out extensively by studying directivity along with FFT analysis of acoustic signature at various distances in the wake of propeller, and by looking at how SPL decays with the distance in the wake of propeller. Moreover, acoustic signature was compared in the near and far fields to see how dominant frequencies change.

#### Acoustic directivity.

**Fig 10:**
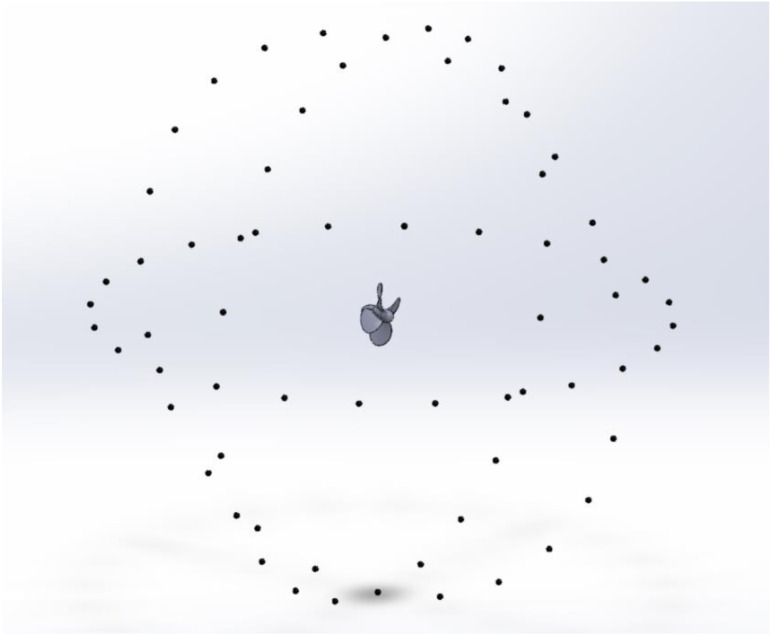
Placement of Virtual hydrophones around propeller across all propeller planes.

Fig 10 illustrates the arrangement of 24 virtual hydrophones in the XY, XZ, and YZ planes at 1.5m diameter to study acoustic directivity. This array enables quantifying the acoustic directivity around the entire propeller geometry.

**Fig 11:**
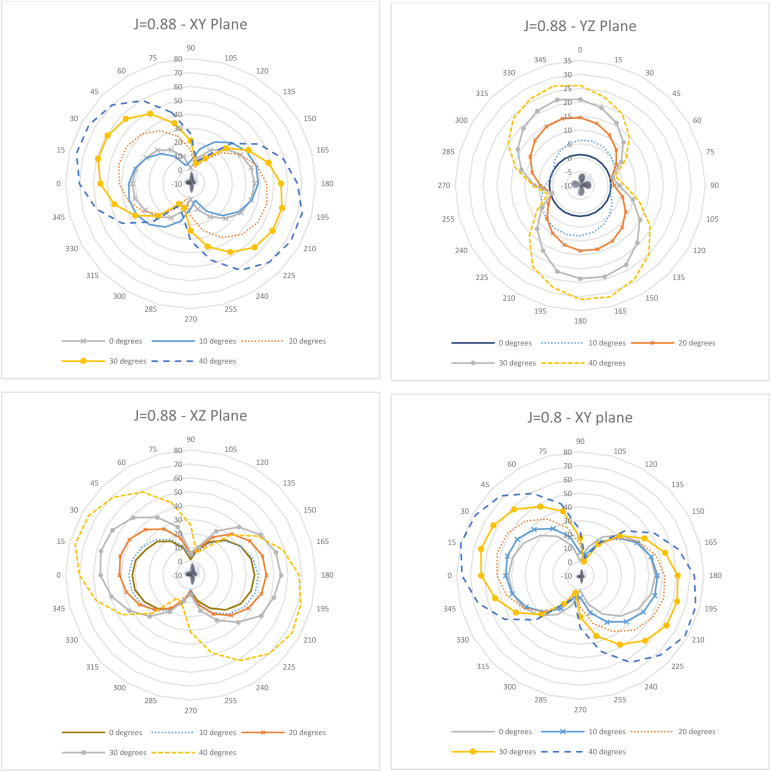
Directivity Plots for J = 0.88 (XY, XZ & YZ plane) and J = 0.8 (XY Plane).

**Fig 12:**
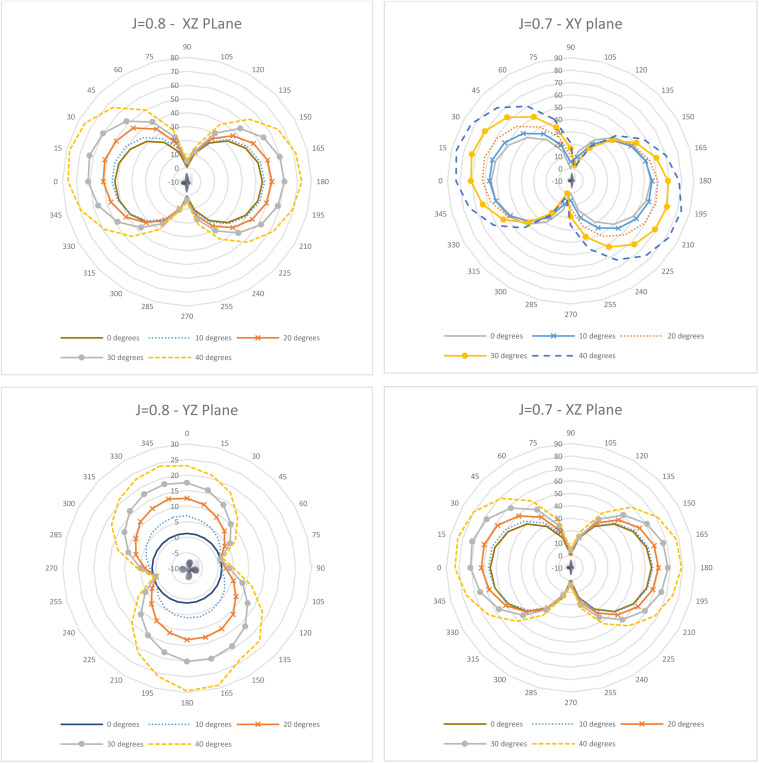
Directivity Plots for J = 0.8 (XZ & YZ plane) and J = 0.7 (XY & XZ Plane).

**Fig 13:**
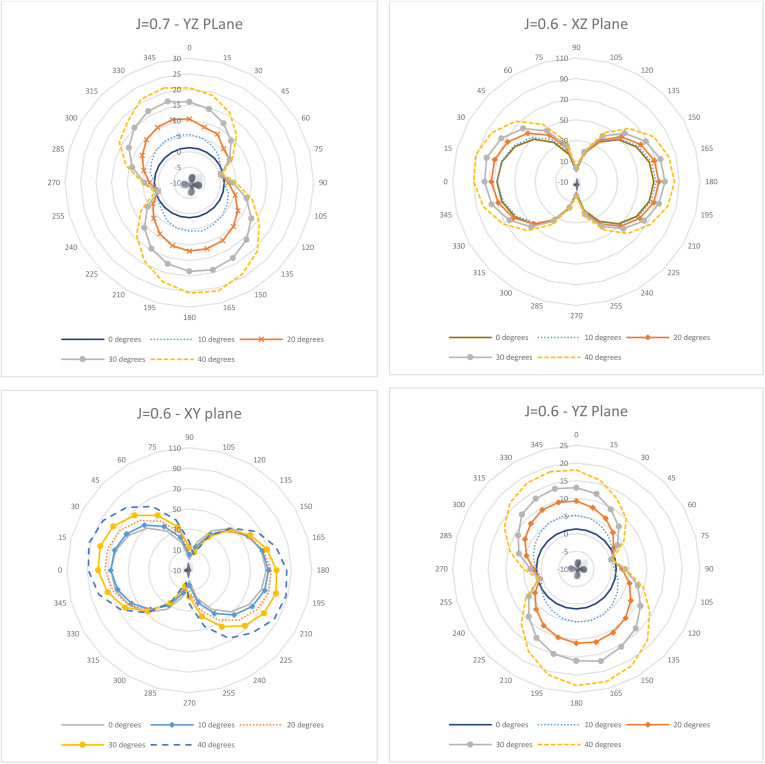
Directivity Plots for J = 0.7 (YZ plane) and J = 0.6 (XY, XZ & YZ Plane).

Directivity plots generated across the three planes for varying J (0.6–0.9) and θ (0–40°) are depicted in Figs 11–13. As observed in Fig 11, the directivity pattern in the XY plane tilts increasingly with rising incidence angle as expected, since the propeller blades slice the incoming flow at an angle. A similar tilting occurs in the YZ plane (Fig 12), while the XZ plane (Fig 11) remains more symmetrical. This indicates the directivity’s sensitivity to the oblique inflow direction.

**Fig 14:**
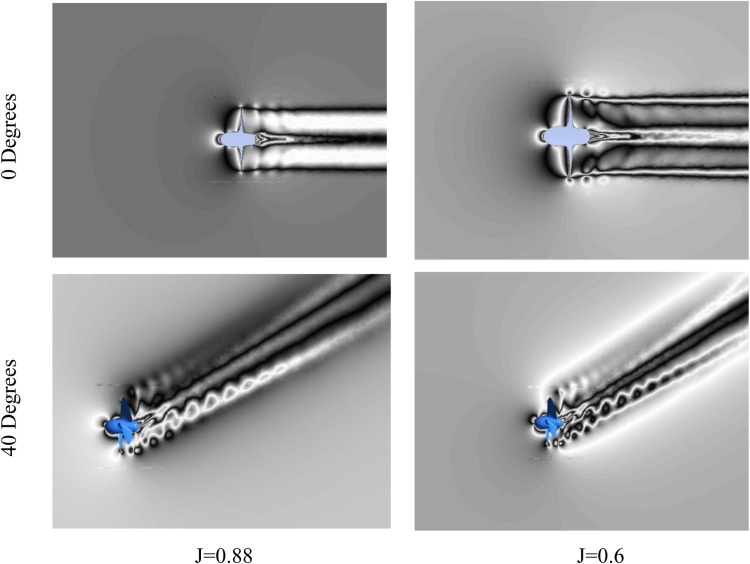
Velocity Contours in Stationary frame across XY plane for J = 0.6 and 0.88.

While the directivity contours maintain a relatively consistent overall directional distribution pattern, there are significant fluctuations in the acoustic pressure magnitude. These variations are closely associated with the incidence angle, as the progressively skewed inflow leads to heightened propeller loading and subsequent flow separation (Figs 11–13). Comparing J = 0.88 and J = 0.6, the average and peak acoustic pressure increase in the XY plane by 17% and 19%, respectively, and by 15–16.5% in the XZ plane. However, a decrease of 18% (average) and 31% (peak) is observed in the YZ plane, attributable to the well-defined slipstream at 0° maintaining intensity better than the separated flow at 40° (Fig 14). This aligns with the pressure contours on the propeller surface in Figs 5–7 and 9. Regarding directivity, acoustic pressure levels tend to be higher at the lower J of 0.6. This phenomenon could be explained as follows: to maintain an equivalent flow velocity at higher advance ratios, the propeller necessitates a higher rotational speed, leading to increased propeller loading and, consequently, heightened noise generation.

The directivity plots show reduced acoustic pressure near the propeller periphery due to slipstream contraction at high RPMs. Propeller rotation creates suction pressure upstream and high pressure downstream, contracting the central slipstream. This induces rapid peripheral inflow to fill the slipstream. Consequently, loading noise coming from the surface of the propeller blades, with preferential directions upstream and downstream (split streams), resulting in lower values on the propeller plane

Fig 14 illustrates velocity distributions in the stationary frame for propeller incidence angles of 0° and 40° across various advance ratios. At 0° incidence, the slipstream downstream of the propeller shows remarkable stability and uniformity, with minimal flow separation evident in the wake. Conversely, at 40° incidence, pronounced flow separation occurs in both the XY and XZ planes. These separated flow structures can propagate within the mainstream flow over considerable distances before dissipation, potentially leading to increased acoustic signatures downstream. Additionally, reducing the advance ratio to J = 0.6 intensifies flow separations within the propeller wake, with the hub vortex showing enhanced strength compared to the J = 0.88 case. These wake dynamics offer valuable insights into variations in near-field pressure fields under different incidences and advance ratios

#### SPL Decay behind propeller.

The decay of sound pressure level (SPL) with distance in the propeller wake was examined for advance ratios of 0.6 and 0.88 at incidence angles of 0° and 40°. The results showed a substantial difference in the SPL increase at 1xBPF when reducing the advance ratio from 0.88 to 0.6, depending on the incidence angle. At 0° incidence, the SPL increased significantly by 18 dB from 96 dB to 114 dB. However, for the 40° case, the SPL increase was much smaller at only 7 dB. This difference can be attributed to the propeller operating closer to optimum efficiency at 0° incidence with lower loading, whereas loading increased substantially with the lower advance ratio, elevating SPL. In contrast, at 40° incidence the propeller experienced considerable flow separation already at J = 0.88, such that lowering the advance ratio had less impact on SPL.

**Fig 15:**
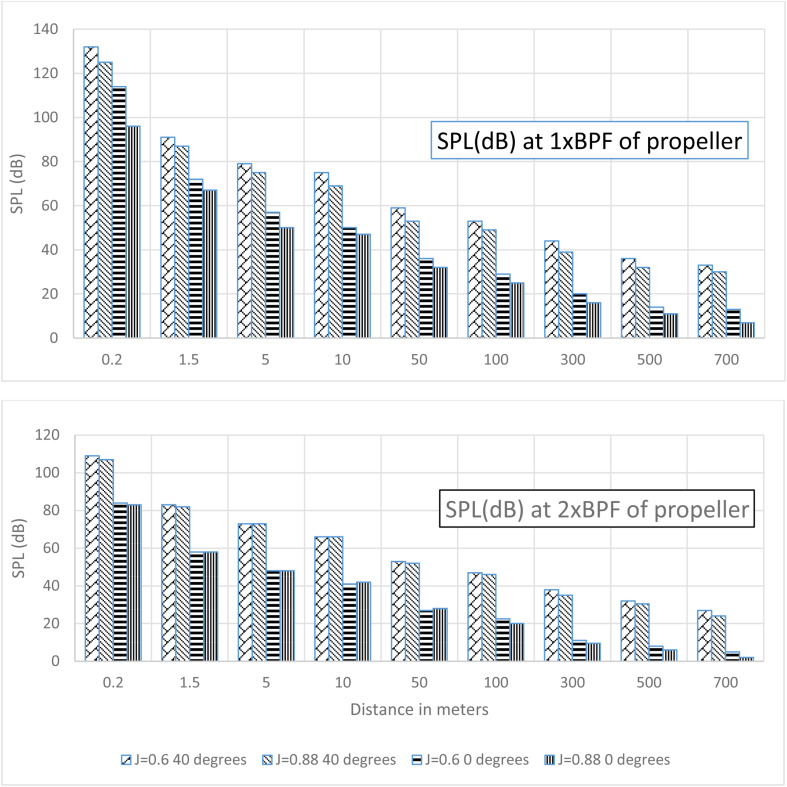
SPL (dB) decay at 1xBPF and 2xBPF for J = 0.6 &0.88 and θ =  0 & 40.

At 40° incidence, the SPL at 2xBPF is much closer in magnitude to 1xBPF and stronger than at 0° incidence. This indicates that secondary sound generation mechanisms play a greater role in higher incidences. Examination of the pressure contours of the propeller at 40° (Fig 5) reveals that two blades experience significantly higher suction and pressure side loads than the other two blades. Analysis of velocity fields in stationary frames at both 0° and 40° incidence in both the XY and XZ planes shows that at 0° the wake is much more stable, and the slipstream of the propeller is clearly defined, whereas in the case of 40° the inflow blades which cut the flow first exhibit higher separation in the wake region behind the propeller. This leads to higher sound amplitudes in the far field, as these flow structures are capable of being carried over greater distances before dissipating. This can be seen from the comparison of SPL versus distance, where zero degrees at J = 0.88 SPL drops to almost 20 dB at 100m, but 40° SPL remains well above 20 dB out to 700m Table 3, Fig 15.

**Table 3:** SPL (dB) decay with distance in the wake of the propeller.

Distance (m)	SPL (dB)							
	0 degrees				40 degrees			
	J = 0.6		J = 0.88		J = 0.6		J = 0.88	
	1xBPF	2xBPF	1xBPF	2xBPF	1xBPF	2xBPF	1xBPF	2xBPF
**0.2**	114	84	96	83	132	109	125	107
**1.5**	72	58	67	58	91	83	87	82
**5**	57	48	50	48	79	73	75	73
**10**	50	41	47	42	75	66	69	66
**50**	36	27	32	28	59	53	53	52
**100**	29	22.5	25	20	53	47	49	46
**300**	20	11	16	9.5	44	38	39	35
**500**	14	8	11	6	36	32	32	30.5
**700**	13	5	7	2	33	27	30	24

#### Acoustic contribution from various parts of the propeller.

**Fig 16:**
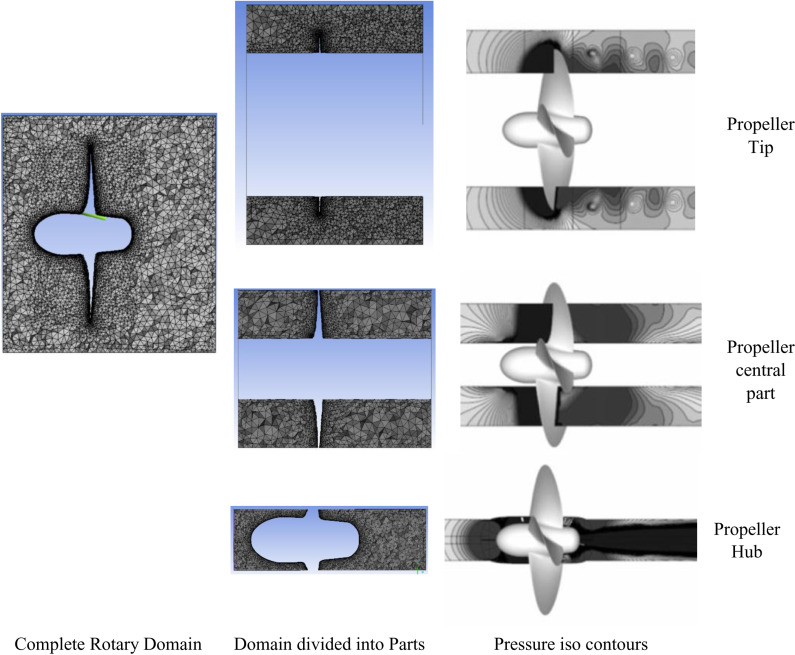
Acoustic Contribution from various parts of the propeller.

The propeller’s rotary domain was divided into three separate regions to account for acoustic contributions from various parts of the propeller, including the Hub, the central portion of the blade, and the Blade Tip. These regions were then individually rotated within a typical global frame of reference to obtain the contribution of each part to the propeller’s acoustic signal (Fig 16).

Results extracted from different segments of the propeller demonstrate that, at a zero-degree angle of attack, the Blade Tip exhibited the most substantial contribution to acoustic pressure, as evidenced in Table 4. It is worth noting that as the angle of attack increased from 0 degrees to 40 degrees, the acoustic pressure values also exhibited an upward trend. Concurrently, there was an approximately 7% increase in the contribution from the Blade Tip. An intriguing observation emerged, indicating a reduction in the contribution from the Hub as the angle of attack increased to higher degrees.

**Table 4:** Acoustic Contribution from Various parts of propeller.

Angle of attack	Percentage Contribution from Propeller Parts		
Propeller Tip	Propeller central part	Propeller Hub
0 Degrees	51	40	9
40 degrees	58	36	6

#### FFT Comparison at Different J and θ.

SPL comparisons were performed for three cases to examine the effect of changes in incidence angle and advance ratio on the frequency distribution of the acoustic signature. The SPL comparisons at various distances reveal that near the propeller, SPL peaks occur at lower frequencies of 1X and 2X BPF (blade passing frequency), which is indicative of dominant propeller tonal noise in this region. However, with increasing distance from the propeller, SPL peaks shift to higher frequencies associated with flow separation phenomena, which are responsible for broadband noise.

**Fig 17:**
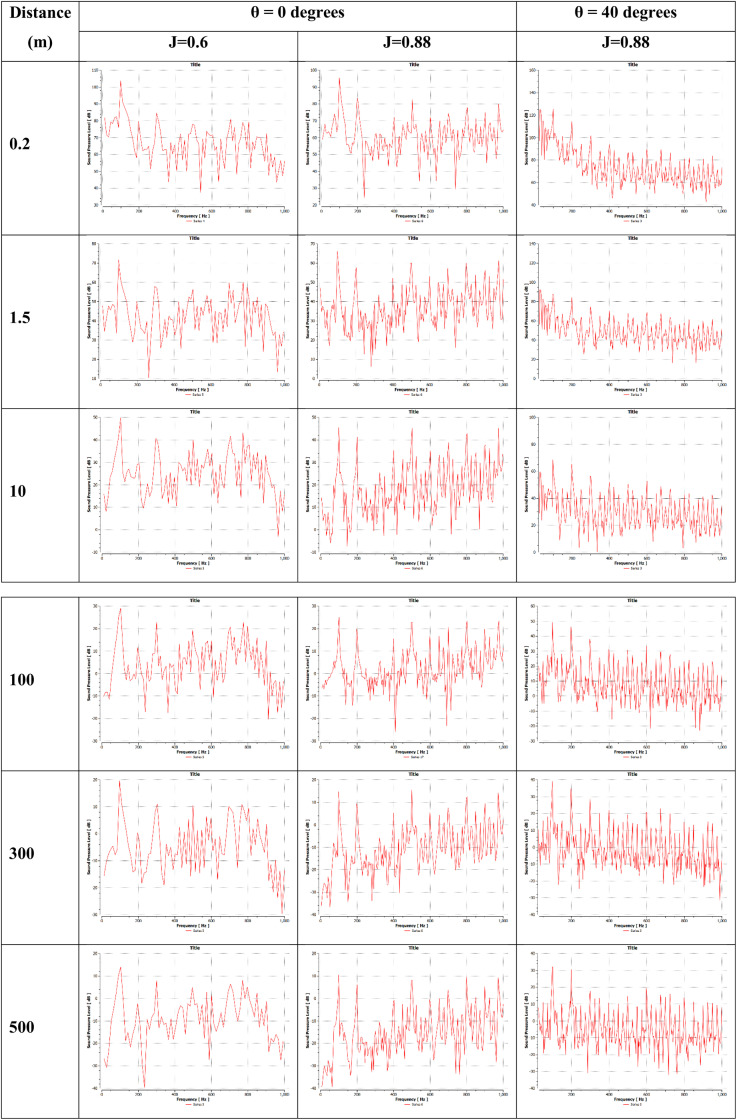
FFT of acoustic signal at J = 0.6 and 0.88 and θ =  0 & 40.

It is observed that by reducing the advance ratio from 0.88 to 0.6, the lower frequency peaks remain dominant over a greater downstream distance in the wake field of the propeller, compared to the higher advance ratio, where higher frequencies dominate at much closer proximity. The lower frequency peaks exhibit significantly greater magnitudes at the higher incidence angle of 40 degrees, indicating increased propeller loading. Furthermore, peaks emerge at frequencies that are integer multiples of the BPF farther downstream from the propeller for the 40 degree case. This suggests flow separations are occurring preferentially at the blade passing rate. A larger number of elevated high-frequency peaks are also observed at 40 degrees relative to 0 degrees, demonstrating a markedly higher flow separation (Fig 17).

Understanding the behavior of marine propellers under oblique inflow conditions holds significant implications across various maritime applications. Primarily, insights gained from such investigations are crucial for maneuvering operations, where vessels frequently encounter non-optimal flow angles during intricate maneuvers such as docking, berthing, or navigating through narrow channels. Additionally, the study of off-design conditions provides invaluable information for real-world scenarios where vessels operate in dynamic environments characterized by changing flow patterns induced by ship motions, waves, or ocean currents. Furthermore, in the realm of submarine operations, where dynamic positioning and manoeuvrability are paramount, comprehending propeller performance under varying flow angles is imperative for ensuring optimal propulsion efficiency and stealth capabilities. From a naval architecture standpoint, optimizing propeller designs for naval vessels necessitates a thorough understanding of propeller behavior in complex flow conditions, enabling the development of efficient and quiet propulsion systems. Finally, research and development efforts aimed at enhancing propeller hydrodynamics and hydroacoustic benefit immensely from studying propellers under diverse operating conditions, leading to advancements in design methodologies, performance predictions, and environmental impact assessments.

## Conclusions

The extensive hydroacoustic investigation explains the sensitivity of propeller performance and noise emissions to advance ratio and incidence angle. The analyses quantify the impacts on loading, efficiency, directivity and frequency characteristics.

Propeller efficiency decreased significantly with rising incidence angle, dropping from 64% to 28% between 0–40° at J = 0.88. A smaller reduction occurred for J = 0.6, potentially because this condition already experienced higher initial loading at 0°. Pressure contours qualitatively showed increasingly asymmetric distributions across blades under higher incidence. This suggests uneven velocities induced across the blades with changing angles of attack.

SPL analysis showed a substantial 18 dB increase in 1xBPF when reducing the advance ratio from 0.88 to 0.6 at 0° angle of attack, compared to a smaller 7 dB rise at 40°. Significant separation occurred at higher angles of attack, intensifying with lower advance ratios. This coincided with elevated 2xBPF levels relative to 1xBPF, indicating augmented secondary sound generation from separation effects. FFT analyses revealed that lower frequency peaks dominated near the propeller, producing a tonal quality. However, higher frequency components from flow separation phenomena emerged at increasing downstream distances and broadened the spectra. Reducing the advance ratio correlated with lower frequencies dominating over a greater extent. Additionally, elevating the incidence angle significantly increased the magnitude of lower components, indicating higher loading. The analysis showed that separated flow disturbances correlated to blade passing frequency played a stronger noise generation role at higher incidences. Evidence suggested turbulence within boundary layers was transported farther before breakdown under elevated incidence. Near the propeller, energy is maximized at the blade pass and rotational frequencies, exhibiting tonal characteristics. However, farther downstream energy is concentrated more at higher frequencies associated with flow phenomena, indicative of broadband noise. Examination of instantaneous signals supported this near-to-far transition, showing sinusoidal waveforms close to the propeller transitioning to irregular, broadband forms at increased downstream distances. These qualitative findings enhance understanding of how loading-dependent propagation transforms propeller noise spectra through influences on separation dynamics.

## References

[1] Bagheri MR, et al. An experimental and numerical prediction of marine propeller noise under cavitating and non-cavitating conditions. Brodogradnja Int J Naval Archit Ocean Eng Res Develop. 2015;66(2):29–45.

[2] Dubbioso G, Muscari R, Di Mascio A. Analysis of a marine propeller operating in oblique flow. Part 2: very high incidence angles. Comput Fluids. 2014;92:56–81.

[3] Krasilnikov V, Zhang Z, Hong F. Analysis of unsteady propeller blade forces by RANS. Proceedings of the First International Symposium on Marine Propulsors. 2009

[4] Shamsi R, Ghassemi H, Molyneux D, Liu P. Numerical hydrodynamic evaluation of propeller (with hub taper) and podded drive in azimuthing conditions. Ocean Eng. 2014;76:121–35. doi: 10.1016/j.oceaneng.2013.10.009

[5] Abbasi A, Ghassemi H, Fadavie M. Hydrodynamic characteristic of the marine propeller in the oblique flow with various current angle by CFD solver. Am J Mar Sci. 2018;6(1):25–9.

[6] Bagheri MR, Seif MS, Mehdigholi H, Yaakob O. Analysis of noise behaviour for marine propellers under cavitating and non-cavitating conditions. Ships Offshore Struct. 2015;12(1):1–8. doi: 10.1080/17445302.2015.1099224

[7] Kim S, Kinnas SA. Numerical prediction of propeller-induced noise in open water and ship behind conditions. Ocean Eng. 2022;261:112122. doi: 10.1016/j.oceaneng.2022.112122

[8] Felli M, Falchi M, Dubbioso G. Tomographic-PIV Survey of the Near-field hydrodynamic and hydroacoustic characteristics of a marine propeller. J Ship Res. 2015;59(04):201–8. doi: 10.5957/jsr.2015.59.4.201

[9] Yao J. Investigation on hydrodynamic performance of a marine propeller in oblique flow by RANS computations. Int J Naval Archit Ocean Eng. 2015;7(1):56–69. doi: 10.1515/ijnaoe-2015-0005

[10] Tani G, Villa D, Gaggero S, Viviani M, Ausonio P, Travi P, et al. Experimental investigation of pressure pulses and radiated noise for two alternative designs of the propeller of a high-speed craft. Ocean Eng. 2017;132:45–69. doi: 10.1016/j.oceaneng.2017.01.015

[11] Ebrahimi A, Razaghian AH, Seif MS, Zahedi F, Nouri-Borujerdi A. A comprehensive study on noise reduction methods of marine propellers and design procedures. Appl Acoust. 2019;150:55–69. doi: 10.1016/j.apacoust.2018.12.004

[12] Felli M, Falchi M, Dubbioso G. Experimental approaches for the diagnostics of hydroacoustic problems in naval propulsion. Ocean Eng. 2015;106 1–19. doi: 10.1016/j.oceaneng.2015.06.049

[13] Ianniello S, Testa C. An overview on the use of the Ffowcs Williams-Hawkings equation for the hydroacoustic analysis of marine propellers. MARINE VIII: proceedings of the VIII International Conference on Computational Methods in Marine Engineering. 2019.

[14] Pennings PC, Bosschers J, Westerweel J, van Terwisga TJC. Dynamics of isolated vortex cavitation. J Fluid Mech. 2015;778:288–313. doi: 10.1017/jfm.2015.379

[15] Ianniello S, Muscari R, Di Mascio A. Ship underwater noise assessment by the Acoustic Analogy part II: Hydroacoustic analysis of a ship scaled model. J Mar Sci Technol. 2014;19(1):52–74. doi: 10.1234/jmst.2014.001

[16] Cianferra M, Petronio A, Armenio V. Non-linear noise from a ship propeller in open sea condition. Ocean Engineering. 2019;191:106474. doi: 10.1016/j.oceaneng.2019.106474

[17] Mousavi B, Rahrovi A, Kheradmand S. Numerical simulation of tonal and broadband hydrodynamic noises of non-cavitating underwater propeller. Polish Mar Res. 2014;21(3):46–53. doi: 10.2478/pomr-2014-0029

[18] Li D-Q, Hallander J, Johansson T. Predicting underwater radiated noise of a full scale ship with model testing and numerical methods. Ocean Eng. 2018;161:121–35. doi: 10.1016/j.oceaneng.2018.03.027

[19] Keller J, Kumar P, Mahesh K. Examination of propeller sound production using large eddy simulation. Phys Rev Fluids. 2018;3(6):064601. doi: 10.1103/physrevfluids.3.064601

[20] Lidtke AK, Lloyd T, Vaz G. Acoustic modelling of a propeller subject to non-uniform inflow. Proceedings of the Sixth International Symposium on Marine Propulsors. 2019.

[21] Wang Y, Göttsche U, Abdel-Maksoud M. Sound Field Properties of Non-Cavitating Marine Propellers. JMSE. 2020;8(11):885. doi: 10.3390/jmse8110885

[22] Dubbioso G, Muscari R, Ortolani F, Di Mascio A. Numerical analysis of marine propellers low frequency noise during maneuvering. Appl Ocean Res. 2021;106:102461. doi: 10.1016/j.apor.2020.102461

